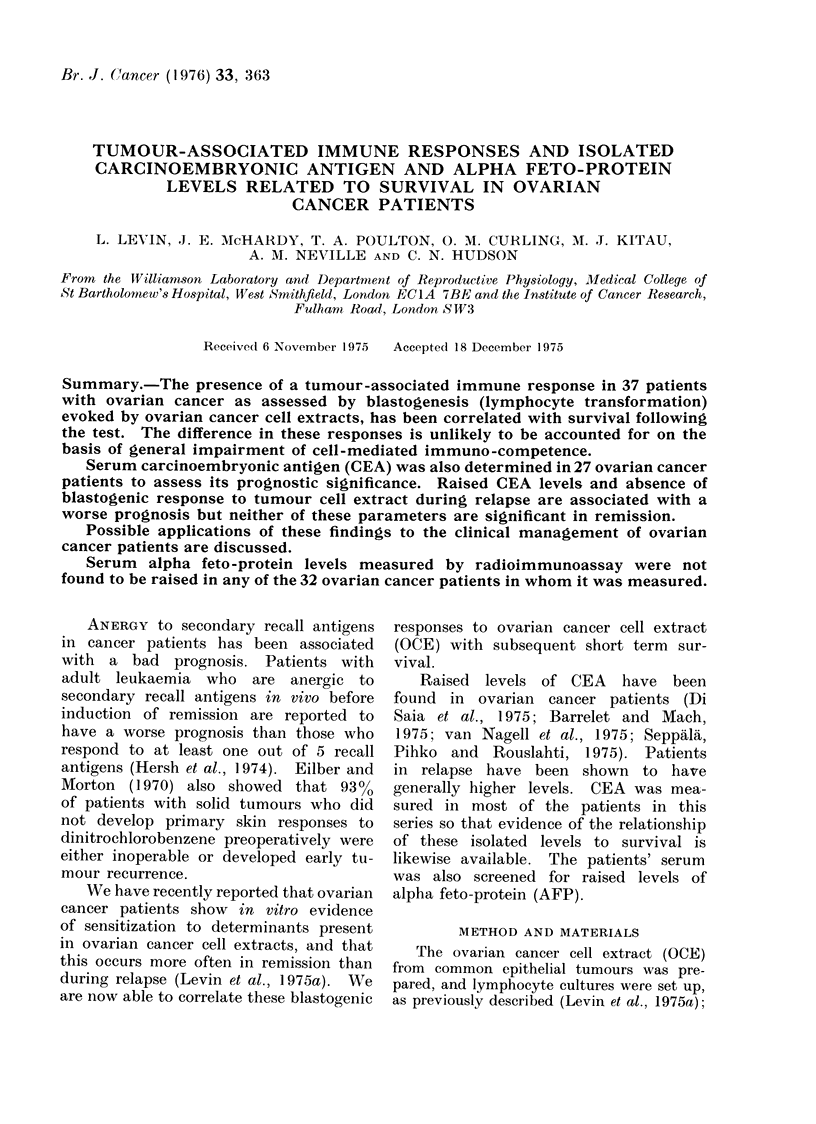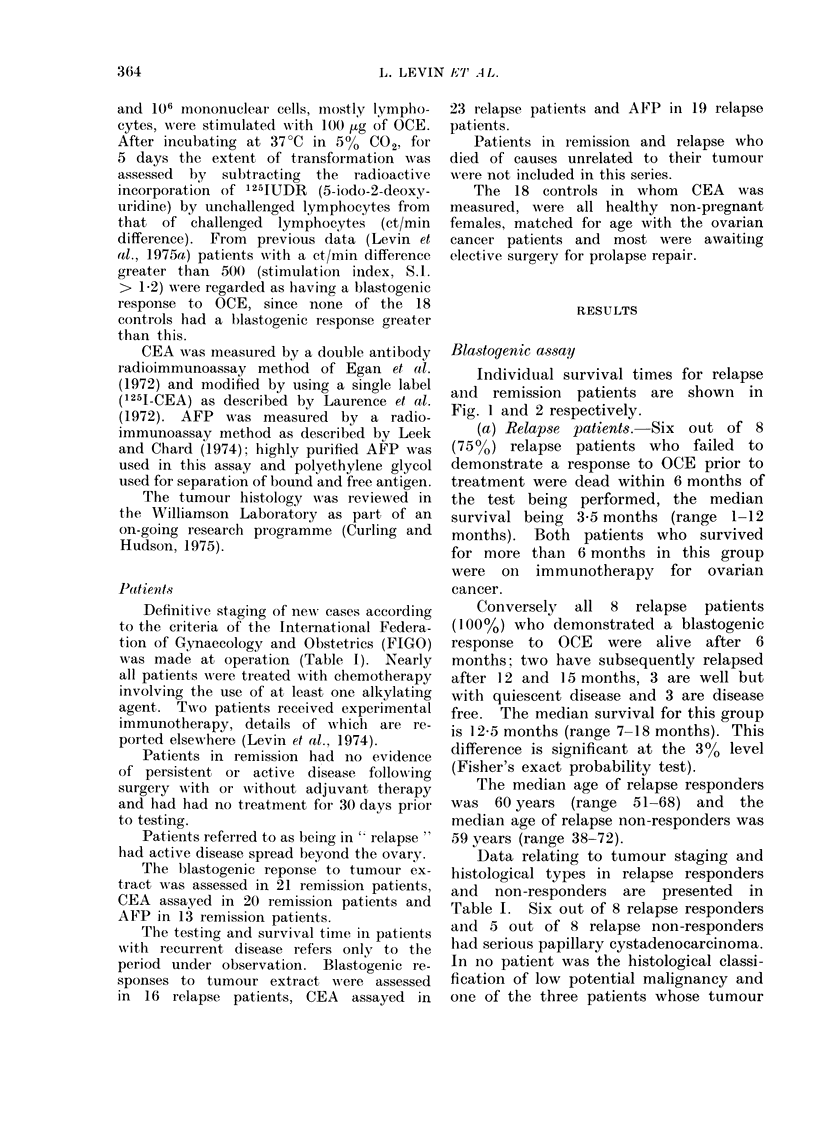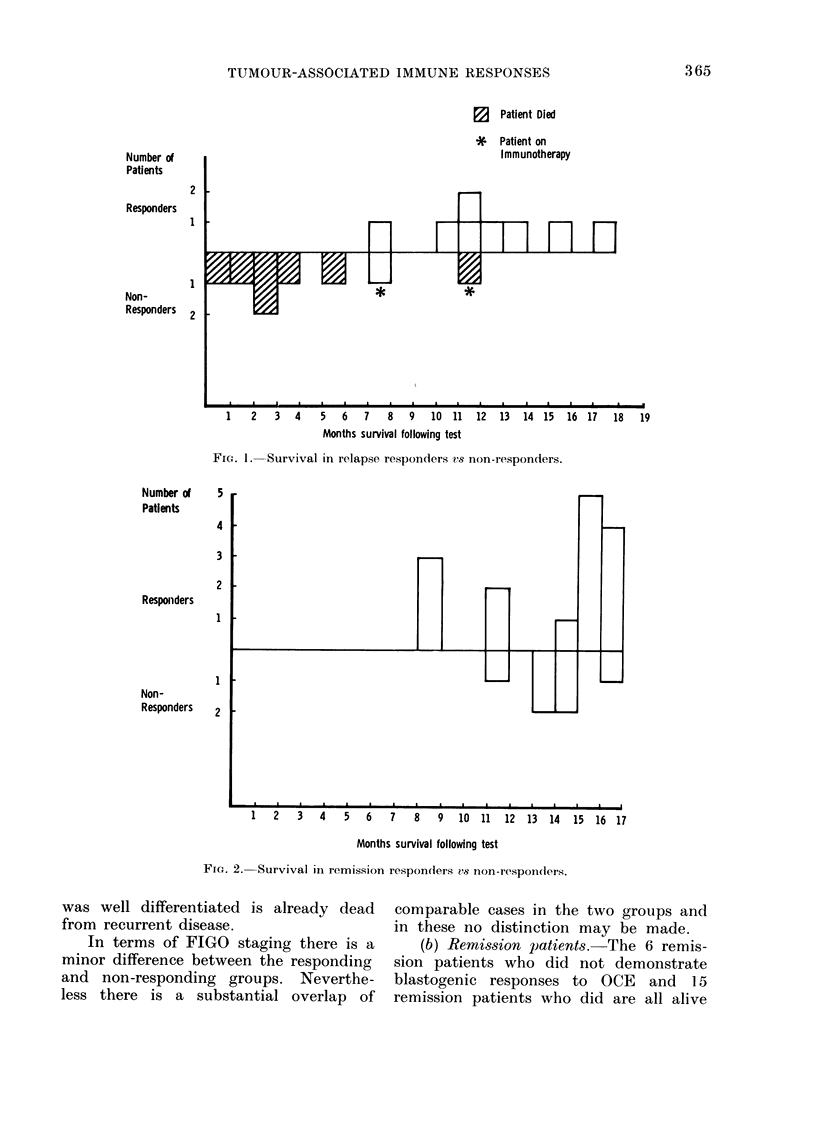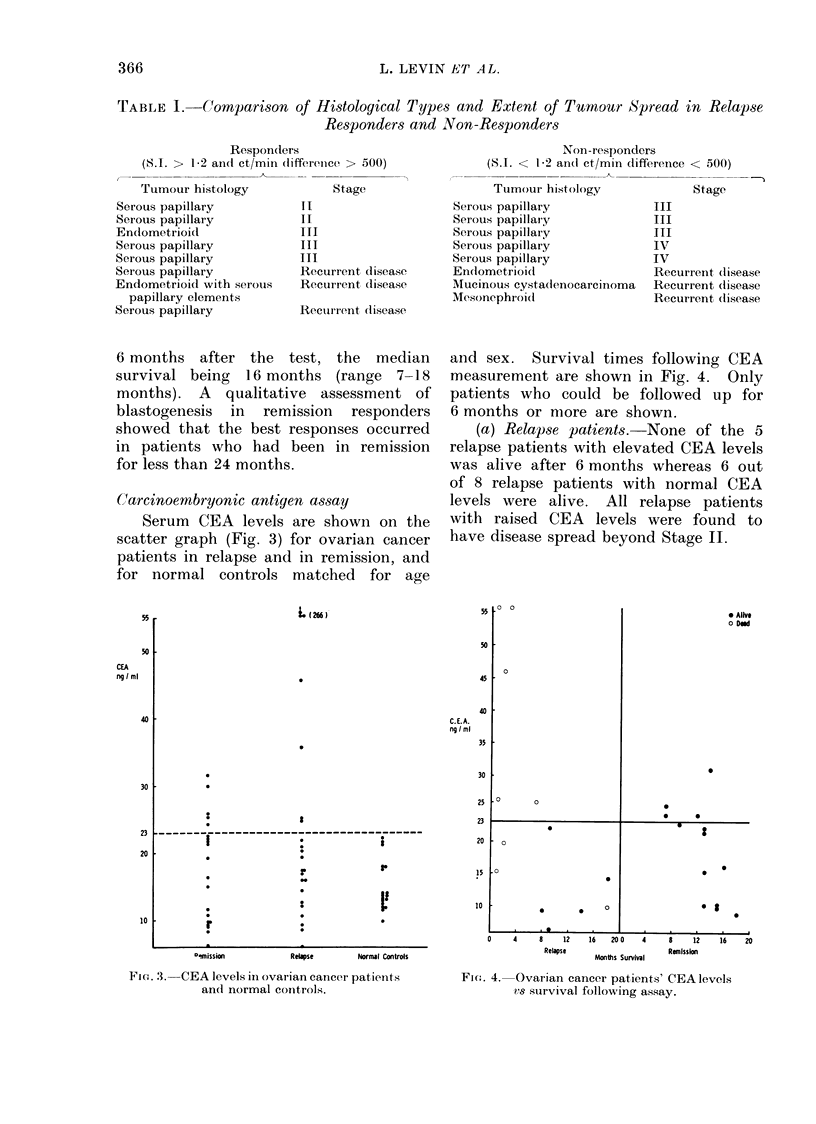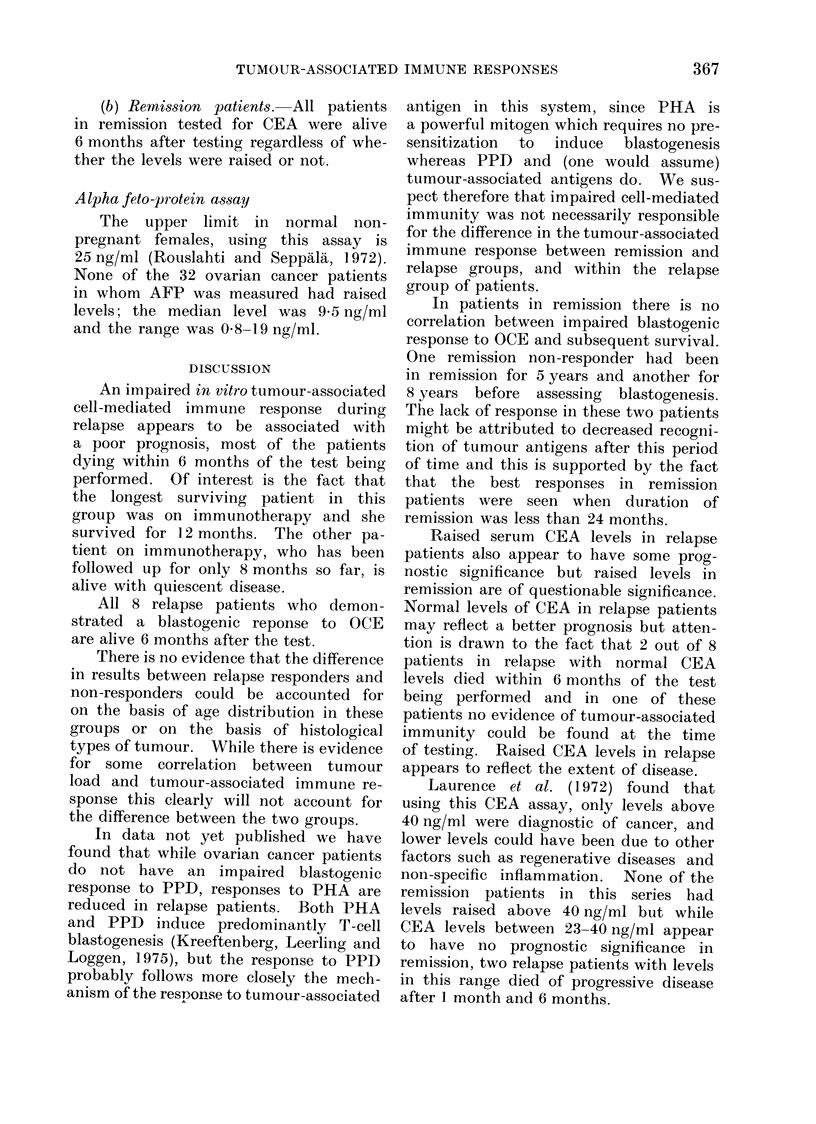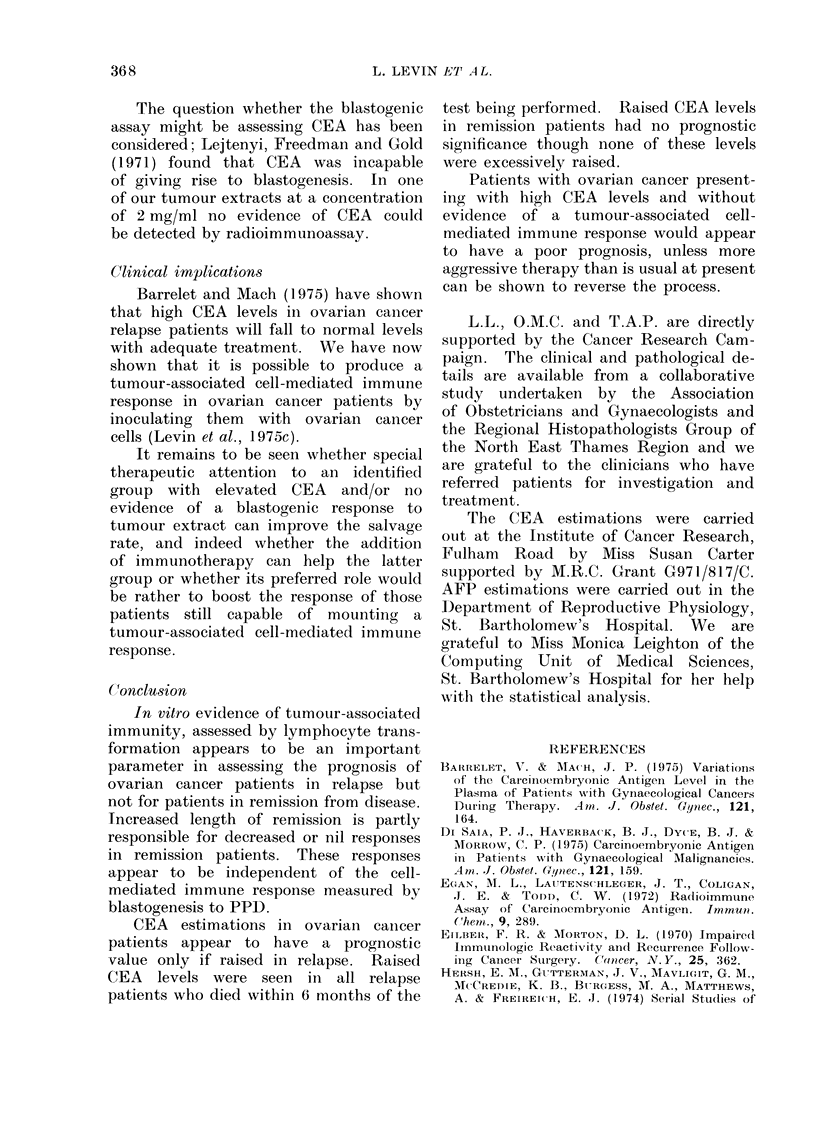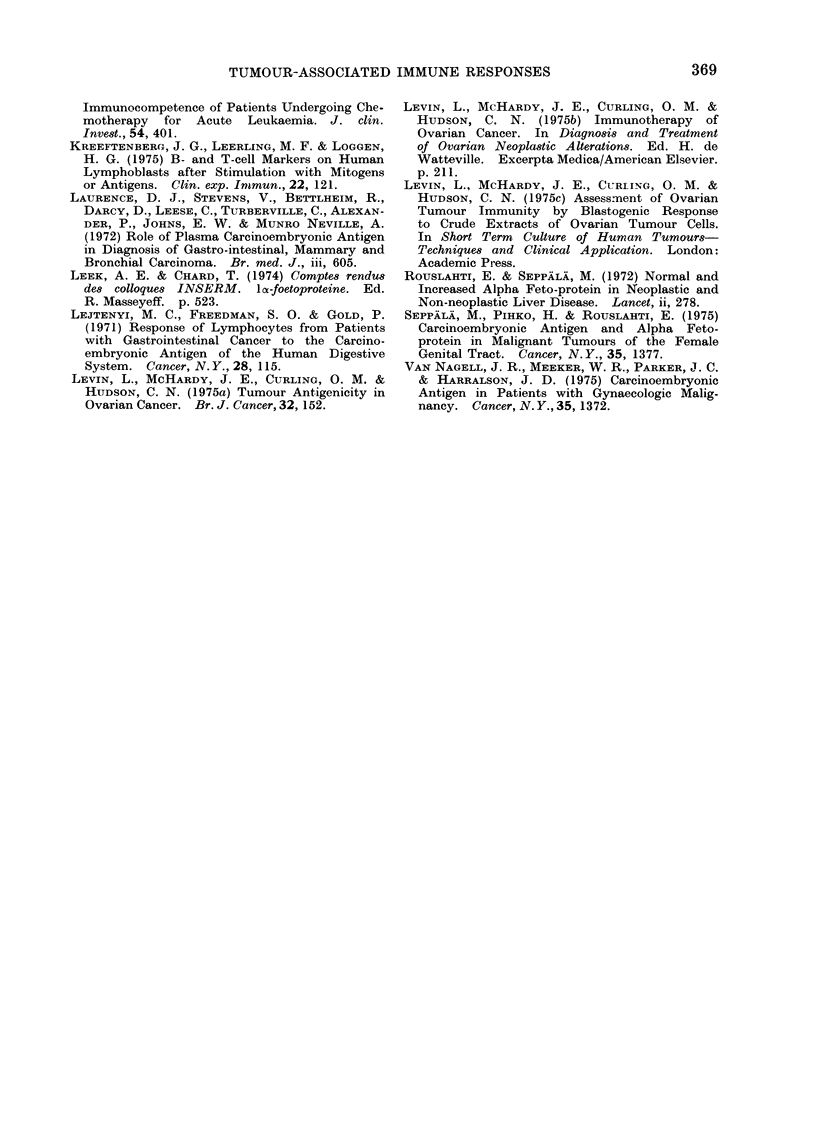# Tumour-associated immune responses and isolated carcinoembryonic antigen and alpha feto-protein levels related to survival in ovarian cancer patients.

**DOI:** 10.1038/bjc.1976.60

**Published:** 1976-04

**Authors:** L. Levin, J. E. McHardy, T. A. Poulton, O. M. Curling, M. J. Kitau, A. M. Neville, C. N. Hudson

## Abstract

The presence of a tumour-associated immune response in 37 patients with ovarian cancer as assessed by blastogenesis (lymphocyte transformation) evoked by ovarian cancer cell extracts, has been correlated with survival following the test. The difference in these responses is unlikely to be accounted for on the basis of general impairment of cell-mediated immuno-competence. Serum carcinoembryonic antigen (CEA) was also determined in 27 ovarian cancer patients to assess its prognostic significance. Raised CEA levels and absence of blastogenic response to tumour cell extract during relapse are associated with a worse prognosis but neither of these parameters are significant in remission. Possible applications of these findings to the clinical management of ovarian cancer patients are discussed. Serum alpha feto-protein levels measured by radioimmunoassay were not found to be raised in any of the 32 ovarian cancer patients in whom it was measured.


Br. J. Cancer (1976) 33, 363

### TUMOUR-ASSOCIATED IMMUNE RESPONSES AND ISOLATED CARCINOEMBRYONIC ANTIGEN AND ALPHA FETO-PROTEIN LEVELS RELATED TO SURVIVAL IN OVARIAN CANCER PATIENTS

L. LEVIN, J. E. McHARDY, T. A. POULTON, O. M. CURLING, M. J. KITAU, A. M. NEVILLE AND C. N. HUDSON

*From the Williamson Laboratory and Department of Reproductive Physiology, Medical College of St Bartholomew's Hospital, West Smithfield, London EC1A 7BE and the Institute of Cancer Research, Fulham Road, London SW3*

Received 6 November 1975 Accepted 18 December 1975

**Summary.—The presence of a tumour-associated immune response in 37 patients with ovarian cancer as assessed by blastogenesis (lymphocyte transformation) evoked by ovarian cancer cell extracts, has been correlated with survival following the test. The difference in these responses is unlikely to be accounted for on the basis of general impairment of cell-mediated immuno-competence.**

**Serum carcinoembryonic antigen (CEA) was also determined in 27 ovarian cancer patients to assess its prognostic significance. Raised CEA levels and absence of blastogenic response to tumour cell extract during relapse are associated with a worse prognosis but neither of these parameters are significant in remission.**

**Possible applications of these findings to the clinical management of ovarian cancer patients are discussed.**

**Serum alpha feto-protein levels measured by radioimmunoassay were not found to be raised in any of the 32 ovarian cancer patients in whom it was measured.**

ANERGY to secondary recall antigens in cancer patients has been associated with a bad prognosis. Patients with adult leukaemia who are anergic to secondary recall antigens *in vivo* before induction of remission are reported to have a worse prognosis than those who respond to at least one out of 5 recall antigens (Hersh *et al.*, 1974). Eilber and Morton (1970) also showed that 93% of patients with solid tumours who did not develop primary skin responses to dinitrochlorobenzene preoperatively were either inoperable or developed early tumour recurrence.

We have recently reported that ovarian cancer patients show *in vitro* evidence of sensitization to determinants present in ovarian cancer cell extracts, and that this occurs more often in remission than during relapse (Levin *et al.*, 1975*a*). We are now able to correlate these blastogenic responses to ovarian cancer cell extract (OCE) with subsequent short term survival.

Raised levels of CEA have been found in ovarian cancer patients (Di Saia *et al.*, 1975; Barrelet and Mach, 1975; van Nagell *et al.*, 1975; Seppälä, Pihko and Rouslahti, 1975). Patients in relapse have been shown to have generally higher levels. CEA was measured in most of the patients in this series so that evidence of the relationship of these isolated levels to survival is likewise available. The patients' serum was also screened for raised levels of alpha feto-protein (AFP).

### METHOD AND MATERIALS

The ovarian cancer cell extract (OCE) from common epithelial tumours was prepared, and lymphocyte cultures were set up, as previously described (Levin *et al.*, 1975*a*); and $10^6$ mononuclear cells, mostly lymphocytes, were stimulated with 100 $\mu$g of OCE. After incubating at 37°C in 5% $CO_2$, for 5 days the extent of transformation was assessed by subtracting the radioactive incorporation of $^{125}$IUDR (5-iodo-2-deoxyuridine) by unchallenged lymphocytes from that of challenged lymphocytes (ct/min difference). From previous data (Levin *et al.*, 1975*a*) patients with a ct/min difference greater than 500 (stimulation index, S.I. $> 1\cdot2$) were regarded as having a blastogenic response to OCE, since none of the 18 controls had a blastogenic response greater than this.

CEA was measured by a double antibody radioimmunoassay method of Egan *et al.* (1972) and modified by using a single label ($^{125}$I-CEA) as described by Laurence *et al.* (1972). AFP was measured by a radioimmunoassay method as described by Leek and Chard (1974); highly purified AFP was used in this assay and polyethylene glycol used for separation of bound and free antigen.

The tumour histology was reviewed in the Williamson Laboratory as part of an on-going research programme (Curling and Hudson, 1975).

*Patients*

Definitive staging of new cases according to the criteria of the International Federation of Gynaecology and Obstetrics (FIGO) was made at operation (Table I). Nearly all patients were treated with chemotherapy involving the use of at least one alkylating agent. Two patients received experimental immunotherapy, details of which are reported elsewhere (Levin *et al.*, 1974).

Patients in remission had no evidence of persistent or active disease following surgery with or without adjuvant therapy and had had no treatment for 30 days prior to testing.

Patients referred to as being in " relapse " had active disease spread beyond the ovary.

The blastogenic reponse to tumour extract was assessed in 21 remission patients, CEA assayed in 20 remission patients and AFP in 13 remission patients.

The testing and survival time in patients with recurrent disease refers only to the period under observation. Blastogenic responses to tumour extract were assessed in 16 relapse patients, CEA assayed in 23 relapse patients and AFP in 19 relapse patients.

Patients in remission and relapse who died of causes unrelated to their tumour were not included in this series.

The 18 controls in whom CEA was measured, were all healthy non-pregnant females, matched for age with the ovarian cancer patients and most were awaiting elective surgery for prolapse repair.

### RESULTS

*Blastogenic assay*

Individual survival times for relapse and remission patients are shown in Fig. 1 and 2 respectively.

(*a*) *Relapse patients.*—Six out of 8 (75%) relapse patients who failed to demonstrate a response to OCE prior to treatment were dead within 6 months of the test being performed, the median survival being 3·5 months (range 1–12 months). Both patients who survived for more than 6 months in this group were on immunotherapy for ovarian cancer.

Conversely all 8 relapse patients (100%) who demonstrated a blastogenic response to OCE were alive after 6 months; two have subsequently relapsed after 12 and 15 months, 3 are well but with quiescent disease and 3 are disease free. The median survival for this group is 12·5 months (range 7–18 months). This difference is significant at the 3% level (Fisher's exact probability test).

The median age of relapse responders was 60 years (range 51–68) and the median age of relapse non-responders was 59 years (range 38–72).

Data relating to tumour staging and histological types in relapse responders and non-responders are presented in Table I. Six out of 8 relapse responders and 5 out of 8 relapse non-responders had serious papillary cystadenocarcinoma. In no patient was the histological classification of low potential malignancy and one of the three patients whose tumour

FIG. 1.—Survival in relapse responders *vs* non-responders.

FIG. 2.—Survival in remission responders *vs* non-responders.

was well differentiated is already dead from recurrent disease.

In terms of FIGO staging there is a minor difference between the responding and non-responding groups. Nevertheless there is a substantial overlap of comparable cases in the two groups and in these no distinction may be made.

(*b*) *Remission patients*.—The 6 remission patients who did not demonstrate blastogenic responses to OCE and 15 remission patients who did are all alive

TABLE I.—*Comparison of Histological Types and Extent of Tumour Spread in Relapse Responders and Non-Responders*

| Responders (S.I. > 1·2 and ct/min difference > 500) Tumour histology | Stage | Non-responders (S.I. < 1·2 and ct/min difference < 500) Tumour histology | Stage |
|---|---|---|---|
| Serous papillary | II | Serous papillary | III |
| Serous papillary | II | Serous papillary | III |
| Endometrioid | III | Serous papillary | III |
| Serous papillary | III | Serous papillary | IV |
| Serous papillary | III | Serous papillary | IV |
| Serous papillary | Recurrent disease | Endometrioid | Recurrent disease |
| Endometrioid with serous papillary elements | Recurrent disease | Mucinous cystadenocarcinoma | Recurrent disease |
| Serous papillary | Recurrent disease | Mesonephroid | Recurrent disease |

6 months after the test, the median survival being 16 months (range 7–18 months). A qualitative assessment of blastogenesis in remission responders showed that the best responses occurred in patients who had been in remission for less than 24 months.

*Carcinoembryonic antigen assay*

Serum CEA levels are shown on the scatter graph (Fig. 3) for ovarian cancer patients in relapse and in remission, and for normal controls matched for age and sex. Survival times following CEA measurement are shown in Fig. 4. Only patients who could be followed up for 6 months or more are shown.

(*a*) *Relapse patients.*—None of the 5 relapse patients with elevated CEA levels was alive after 6 months whereas 6 out of 8 relapse patients with normal CEA levels were alive. All relapse patients with raised CEA levels were found to have disease spread beyond Stage II.

FIG. 3.—CEA levels in ovarian cancer patients and normal controls.

FIG. 4.—Ovarian cancer patients' CEA levels *vs* survival following assay.

(*b*) *Remission patients.*—All patients in remission tested for CEA were alive 6 months after testing regardless of whether the levels were raised or not.

*Alpha feto-protein assay*

The upper limit in normal non-pregnant females, using this assay is 25 ng/ml (Rouslahti and Seppälä, 1972). None of the 32 ovarian cancer patients in whom AFP was measured had raised levels; the median level was 9·5 ng/ml and the range was 0·8–19 ng/ml.

### DISCUSSION

An impaired *in vitro* tumour-associated cell-mediated immune response during relapse appears to be associated with a poor prognosis, most of the patients dying within 6 months of the test being performed. Of interest is the fact that the longest surviving patient in this group was on immunotherapy and she survived for 12 months. The other patient on immunotherapy, who has been followed up for only 8 months so far, is alive with quiescent disease.

All 8 relapse patients who demonstrated a blastogenic reponse to OCE are alive 6 months after the test.

There is no evidence that the difference in results between relapse responders and non-responders could be accounted for on the basis of age distribution in these groups or on the basis of histological types of tumour. While there is evidence for some correlation between tumour load and tumour-associated immune response this clearly will not account for the difference between the two groups.

In data not yet published we have found that while ovarian cancer patients do not have an impaired blastogenic response to PPD, responses to PHA are reduced in relapse patients. Both PHA and PPD induce predominantly T-cell blastogenesis (Kreeftenberg, Leerling and Loggen, 1975), but the response to PPD probably follows more closely the mechanism of the response to tumour-associated antigen in this system, since PHA is a powerful mitogen which requires no presensitization to induce blastogenesis whereas PPD and (one would assume) tumour-associated antigens do. We suspect therefore that impaired cell-mediated immunity was not necessarily responsible for the difference in the tumour-associated immune response between remission and relapse groups, and within the relapse group of patients.

In patients in remission there is no correlation between impaired blastogenic response to OCE and subsequent survival. One remission non-responder had been in remission for 5 years and another for 8 years before assessing blastogenesis. The lack of response in these two patients might be attributed to decreased recognition of tumour antigens after this period of time and this is supported by the fact that the best responses in remission patients were seen when duration of remission was less than 24 months.

Raised serum CEA levels in relapse patients also appear to have some prognostic significance but raised levels in remission are of questionable significance. Normal levels of CEA in relapse patients may reflect a better prognosis but attention is drawn to the fact that 2 out of 8 patients in relapse with normal CEA levels died within 6 months of the test being performed and in one of these patients no evidence of tumour-associated immunity could be found at the time of testing. Raised CEA levels in relapse appears to reflect the extent of disease.

Laurence *et al.* (1972) found that using this CEA assay, only levels above 40 ng/ml were diagnostic of cancer, and lower levels could have been due to other factors such as regenerative diseases and non-specific inflammation. None of the remission patients in this series had levels raised above 40 ng/ml but while CEA levels between 23–40 ng/ml appear to have no prognostic significance in remission, two relapse patients with levels in this range died of progressive disease after 1 month and 6 months.

The question whether the blastogenic assay might be assessing CEA has been considered; Lejtenyi, Freedman and Gold (1971) found that CEA was incapable of giving rise to blastogenesis. In one of our tumour extracts at a concentration of 2 mg/ml no evidence of CEA could be detected by radioimmunoassay.

*Clinical implications*

Barrelet and Mach (1975) have shown that high CEA levels in ovarian cancer relapse patients will fall to normal levels with adequate treatment. We have now shown that it is possible to produce a tumour-associated cell-mediated immune response in ovarian cancer patients by inoculating them with ovarian cancer cells (Levin *et al.*, 1975*c*).

It remains to be seen whether special therapeutic attention to an identified group with elevated CEA and/or no evidence of a blastogenic response to tumour extract can improve the salvage rate, and indeed whether the addition of immunotherapy can help the latter group or whether its preferred role would be rather to boost the response of those patients still capable of mounting a tumour-associated cell-mediated immune response.

*Conclusion*

*In vitro* evidence of tumour-associated immunity, assessed by lymphocyte transformation appears to be an important parameter in assessing the prognosis of ovarian cancer patients in relapse but not for patients in remission from disease. Increased length of remission is partly responsible for decreased or nil responses in remission patients. These responses appear to be independent of the cell-mediated immune response measured by blastogenesis to PPD.

CEA estimations in ovarian cancer patients appear to have a prognostic value only if raised in relapse. Raised CEA levels were seen in all relapse patients who died within 6 months of the test being performed. Raised CEA levels in remission patients had no prognostic significance though none of these levels were excessively raised.

Patients with ovarian cancer presenting with high CEA levels and without evidence of a tumour-associated cell-mediated immune response would appear to have a poor prognosis, unless more aggressive therapy than is usual at present can be shown to reverse the process.

L.L., O.M.C. and T.A.P. are directly supported by the Cancer Research Campaign. The clinical and pathological details are available from a collaborative study undertaken by the Association of Obstetricians and Gynaecologists and the Regional Histopathologists Group of the North East Thames Region and we are grateful to the clinicians who have referred patients for investigation and treatment.

The CEA estimations were carried out at the Institute of Cancer Research, Fulham Road by Miss Susan Carter supported by M.R.C. Grant G971/817/C. AFP estimations were carried out in the Department of Reproductive Physiology, St. Bartholomew's Hospital. We are grateful to Miss Monica Leighton of the Computing Unit of Medical Sciences, St. Bartholomew's Hospital for her help with the statistical analysis.

REFERENCES

Barrelet, V. & Mach, J. P. (1975) Variations of the Carcinoembryonic Antigen Level in the Plasma of Patients with Gynaecological Cancers During Therapy. *Am. J. Obstet. Gynec.*, **121**, 164.

Di Saia, P. J., Haverback, B. J., Dyce, B. J. & Morrow, C. P. (1975) Carcinoembryonic Antigen in Patients with Gynaecological Malignancies. *Am. J. Obstet. Gynec.*, **121**, 159.

Egan, M. L., Lautenschleger, J. T., Coligan, J. E. & Todd, C. W. (1972) Radioimmune Assay of Carcinoembryonic Antigen. *Immun. Chem.*, **9**, 289.

Eilber, F. R. & Morton, D. L. (1970) Impaired Immunologic Reactivity and Recurrence Following Cancer Surgery. *Cancer, N.Y.*, **25**, 362.

Hersh, E. M., Gutterman, J. V., Mavligit, G. M., McCredie, K. B., Burgess, M. A., Matthews, A. & Freireich, E. J. (1974) Serial Studies of

Immunocompetence of Patients Undergoing Chemotherapy for Acute Leukaemia. *J. clin. Invest.*, **54**, 401.

KREEFTENBERG, J. G., LEERLING, M. F. & LOGGEN, H. G. (1975) B- and T-cell Markers on Human Lymphoblasts after Stimulation with Mitogens or Antigens. *Clin. exp. Immun.*, **22**, 121.

LAURENCE, D. J., STEVENS, V., BETTLHEIM, R., DARCY, D., LEESE, C., TURBERVILLE, C., ALEXANDER, P., JOHNS, E. W. & MUNRO NEVILLE, A. (1972) Role of Plasma Carcinoembryonic Antigen in Diagnosis of Gastro-intestinal, Mammary and Bronchial Carcinoma. *Br. med. J.*, iii, 605.

LEEK, A. E. & CHARD, T. (1974) *Comptes rendus des colloques INSERM. 1α-foetoproteine.* Ed. R. Masseyeff. p. 523.

LEJTENYI, M. C., FREEDMAN, S. O. & GOLD, P. (1971) Response of Lymphocytes from Patients with Gastrointestinal Cancer to the Carcinoembryonic Antigen of the Human Digestive System. *Cancer, N.Y.*, **28**, 115.

LEVIN, L., MCHARDY, J. E., CURLING, O. M. & HUDSON, C. N. (1975*a*) Tumour Antigenicity in Ovarian Cancer. *Br. J. Cancer*, **32**, 152.

LEVIN, L., MCHARDY, J. E., CURLING, O. M. & HUDSON, C. N. (1975*b*) Immunotherapy of Ovarian Cancer. In *Diagnosis and Treatment of Ovarian Neoplastic Alterations.* Ed. H. de Watteville. Excerpta Medica/American Elsevier. p. 211.

LEVIN, L., MCHARDY, J. E., CURLING, O. M. & HUDSON, C. N. (1975*c*) Assessment of Ovarian Tumour Immunity by Blastogenic Response to Crude Extracts of Ovarian Tumour Cells. In *Short Term Culture of Human Tumours—Techniques and Clinical Application.* London: Academic Press.

ROUSLAHTI, E. & SEPPÄLÄ, M. (1972) Normal and Increased Alpha Feto-protein in Neoplastic and Non-neoplastic Liver Disease. *Lancet*, ii, 278.

SEPPÄLÄ, M., PIHKO, H. & ROUSLAHTI, E. (1975) Carcinoembryonic Antigen and Alpha Fetoprotein in Malignant Tumours of the Female Genital Tract. *Cancer, N.Y.*, **35**, 1377.

VAN NAGELL, J. R., MEEKER, W. R., PARKER, J. C. & HARRALSON, J. D. (1975) Carcinoembryonic Antigen in Patients with Gynaecologic Malignancy. *Cancer, N.Y.*, **35**, 1372.